# Activation of *Anopheles stephensi* Pantothenate Kinase and Coenzyme A Biosynthesis Reduces Infection with Diverse *Plasmodium* Species in the Mosquito Host

**DOI:** 10.3390/biom11060807

**Published:** 2021-05-29

**Authors:** Raquel M. Simão-Gurge, Neha Thakre, Jessica Strickland, Jun Isoe, Lillian R. Delacruz, Brandi K. Torrevillas, Anna M. Rodriguez, Michael A. Riehle, Shirley Luckhart

**Affiliations:** 1 Department of Entomology, Plant Pathology and Nematology, University of Idaho, Moscow, ID 83843, USA; rsimaogurge@uidaho.edu (R.M.S.-G.); jstrickland@uidaho.edu (J.S.); bktorrevillas@uidaho.edu (B.K.T.); amrodriguez@uidaho.edu (A.M.R.); 2 Department of Entomology, University of Arizona, Tucson, AZ 85721, USA; nthakre@arizona.edu (N.T.); jisoe@ag.arizona.edu (J.I.); lrdelacruz@email.arizona.edu (L.R.D.); mriehle@ag.arizona.edu (M.A.R.); 3 Department of Biological Sciences, University of Idaho, Moscow, ID 83843, USA

**Keywords:** coenzyme A, CoA, pantothenate kinase, PanK, *Anopheles stephensi*, midgut, *Plasmodium falciparum*, *Plasmodium yoelii*, malaria, small molecules, PZ-2891, compound 7

## Abstract

Malaria parasites require pantothenate from both human and mosquito hosts to synthesize coenzyme A (CoA). Specifically, mosquito-stage parasites cannot synthesize pantothenate de novo or take up preformed CoA from the mosquito host, making it essential for the parasite to obtain pantothenate from mosquito stores. This makes pantothenate utilization an attractive target for controlling sexual stage malaria parasites in the mosquito. CoA is synthesized from pantothenate in a multi-step pathway initiated by the enzyme pantothenate kinase (PanK). In this work, we manipulated *A. stephensi* PanK activity and assessed the impact of mosquito PanK activity on the development of two malaria parasite species with distinct genetics and life cycles: the human parasite *Plasmodium falciparum* and the mouse parasite *Plasmodium yoelii yoelii* 17XNL. We identified two putative *A. stephensi* PanK isoforms encoded by a single gene and expressed in the mosquito midgut. Using both RNAi and small molecules with reported activity against human PanK, we confirmed that *A. stephensi* PanK manipulation was associated with corresponding changes in midgut CoA levels. Based on these findings, we used two small molecule modulators of human PanK activity (PZ-2891, compound 7) at reported and ten-fold EC_50_ doses to examine the effects of manipulating *A. stephensi* PanK on malaria parasite infection success. Our data showed that oral provisioning of 1.3 nM and 13 nM PZ-2891 increased midgut CoA levels and significantly decreased infection success for both *Plasmodium* species. In contrast, oral provisioning of 62 nM and 620 nM compound 7 decreased CoA levels and significantly increased infection success for both *Plasmodium* species. This work establishes the *A. stephensi* CoA biosynthesis pathway as a potential target for broadly blocking malaria parasite development in anopheline hosts. We envision this strategy, with small molecule PanK modulators delivered to mosquitoes via attractive bait stations, working in concert with deployment of parasite-directed novel pantothenamide drugs to block parasite infection in the human host. In mosquitoes, depletion of pantothenate through manipulation to increase CoA biosynthesis is expected to negatively impact *Plasmodium* survival by starving the parasite of this essential nutrient. This has the potential to kill both wild type parasites and pantothenamide-resistant parasites that could develop under pantothenamide drug pressure if these compounds are used as future therapeutics for human malaria.

## 1. Introduction

Malaria remains a devastating disease and significant efforts have focused on developing novel therapeutics, vaccines, and mosquito-targeted strategies to eliminate human infection and disrupt parasite transmission. During development, malaria parasites must acquire essential host resources that they are unable to synthesize for themselves. Blocking parasite access to these resources, via altered host synthesis or by preventing parasite uptake, can be lethal and is the basis for novel anti-*Plasmodium* strategies being explored for human treatment. Among the host nutrients required by malaria parasites is pantothenate or vitamin B5. *Plasmodium* spp. cannot synthesize pantothenate de novo and, therefore, must acquire pantothenate from the vertebrate and mosquito hosts for survival [1,2]. Malaria parasites, like both of their hosts, convert pantothenate to coenzyme A (CoA), a critical co-factor for metabolism, via the initial enzyme in the CoA biosynthesis pathway, pantothenate kinase (PanK).

Once pantothenate enters the CoA biosynthesis pathway PanK initiates its rapid, multi-step conversion to CoA, with only the metabolite 4′-phosphopantetheine found at detectable levels [3]. Mammalian genomes encode four PanK isoforms, including PanK1**α**, PanK1**β**, PanK2, and PanK3. Four additional pathway enzymes, including 4′-phospho-pantothenoylcysteine synthetase (PPCS), 4′-phosphopanto-thenoylcysteine decarboxylase (PPCDC), 4′- phosphopantetheine adenylyltransferase (PPAT), and dephospho-CoA kinase (DPCK) process intermediates of the pathway [2]. The genome of the aggressive biter and invasive malaria vector *Anopheles stephensi* [4] encodes individual orthologs for PanK and other pathway gene products, with PPAT and DPCK encoded by a single *A. stephensi* gene as in many other eukaryotes [5].

Studies with both human and mouse malaria parasites highlight the importance of pantothenate-CoA metabolism across both asexual and sexual life stages [2,6,7,8,9]. For example, Tjhin et al. [8] showed that *Plasmodium falciparum* PanK1 is essential for asexual parasite growth. Notably, pantothenate analogs, termed pantothenamides, capable of disrupting pantothenate metabolism and CoA utilization in *P. falciparum* were also gametocytocidal [9], extending the requirement of pantothenate-CoA to sexual stage parasite development. In recent work, Tjhin et al. demonstrated that *P. falciparum* PanK1 and PanK2 form a protein complex with a single complete active site perhaps regulated by a bound dimer of *P. falciparum* 14-3-3I [7]. In contrast to *P. falciparum*, *P. yoelii* PanK1 or PanK2 knockouts successfully completed asexual development and gametocyte formation in infected mice [6], affirming that some *Plasmodium* spp. can utilize alternative host precursors for CoA synthesis. However, parasites with a double knockout of *P. yoelii* PanK1/2 were severely deficient in ookinete and oocyst development and unable to produce sporozoites in *A. stephensi*, indicating that, like *P. falciparum* PanK1, *P. yoelii* PanK1/2 are required for the completion of sexual stage development [6]. In a subsequent study, Hart et al. [2] confirmed that mosquito-stage parasites cannot acquire preformed CoA from the mosquito host, and thus are entirely dependent on the uptake of the pantothenate precursor from the mosquito.

To demonstrate the critical requirement of pantothenate by malaria parasites, pantothenamides have been developed with the goal of disrupting parasite CoA/acetyl-CoA biology via the formation of non-functional metabolites [1,8,9,10,11]. A novel pantothenamide suppressed *P. falciparum* parasitemia in a humanized mouse model [9]. In these comprehensive studies, the authors selected pantothenamide-resistant *P. falciparum* as a strategy to reveal the anti-parasite mode of action and to predict drug modifications that might prevent the development of pantothenamide resistance [9]. Resistant parasites grew slowly and were deficient in CoA and acetyl-CoA, but generated oocyst infections in *A. stephensi* comparable to normal parasites [9], raising concerns about the spread of resistance and loss of a new drug strategy if this were to occur.

These concerns supported our focus on the mosquito host as a potential secondary target for manipulation of parasite access to host pantothenate to block transmission of both susceptible parasites and those that might develop pantothenamide resistance. In earlier work, we demonstrated that *P. falciparum* infection of *A. stephensi* increased activation of c-Jun N-terminal kinase (JNK) signaling in the mosquito midgut and, conversely, that inhibition of JNK signaling activation was associated with increased CoA biosynthesis and expression of *A. stephensi* PanK in the mosquito midgut [12]. We proposed that parasite-induced JNK signaling would benefit the parasite by decreasing competition with the mosquito host for pantothenate, with studies showing that JNK signaling inhibition was also associated with upregulated CoA synthesis in the *A. stephensi* midgut and increased resistance to *P. falciparum* infection [12]. These studies suggested for the first time that shifts in pantothenate-CoA metabolism in mosquito midgut might adversely affect sexual stage parasite development by starving the parasite of pantothenate [12].

Here, we present data to test this novel hypothesis, affirming that *A. stephensi* PanK activity controls CoA levels in the midgut epithelium and that mosquito PanK-catalyzed CoA biosynthesis in the midgut can be controlled via orally available small molecules predicted to regulate *A. stephensi* PanK by the same allosteric mechanism observed for human PanK. Importantly, provisioning of these PanK-modulating small molecules that increased or decreased midgut CoA levels predictably decreased and increased, respectively, the infection of *A. stephensi* with both *P. falciparum* and the mouse parasite *Plasmodium yoelii yoelli* 17XNL. These findings demonstrate that small molecules can be delivered orally to *A. stephensi* in support of a longer-term goal of reducing mosquito-stage infection and development of markedly different parasite species that share a genus-level dependency on mosquito pantothenate for survival.

## 2. Materials and Methods

### 2.1. Chemicals and Reagents

Protease inhibitor solution (Complete™ Mini Protease Inhibitor Cocktail tablets; Sigma, St. Louis, MO, USA) was prepared by dissolving 1 tablet in 5 mL of 1X phosphate-buffered saline (PBS). Small molecule PanK modulators PZ-2891 (MedKoo Biosciences, Morrisville, NC, USA) and compound 7 (Calbiochem, San Diego, CA, USA) were purchased for these studies. *Plasmodium falciparum* NF54 was maintained in RPMI-1640 medium (Sigma, St. Louis, MO, USA) supplemented with HEPES, L-glutamine, hypoxanthine (Thermo Scientific, Waltham, MA, USA) and DL-Lactic acid (Thermo Scientific, Waltham, MA, USA) with 10% (*v*/*v*) human serum and 4.0–6.0% washed type O+ red blood cells (RBCs, Interstate Blood Bank, Memphis, TN, USA). Propidium iodide (Sigma-Aldrich, St. Louis, MO, USA) was diluted to a final concentration of 10 µg/mL in PBS [13]. An *A. stephensi* PanK custom polyclonal antibody was generated against a 16 amino acid peptide (KALFLEHEGYFGAVGC) at the C-terminus of *A. stephensi* PanK conjugated to KLH and inoculated into rabbits following the Proteintech 102-day immunization protocol (Proteintech, Rosemont, IL, USA). The neat serum was column affinity-purified against the *A. stephensi* PanK peptide.

### 2.2. A. stephensi Rearing and Maintenance

*A. stephensi* (Indian strain) was maintained at 27 °C and 80% humidity with light/dark cycling. Adult mosquitoes were provided with a 10% sucrose solution ad libitum. For colony maintenance, adult female *A. stephensi* were provisioned with bovine blood (University of Arizona Food Products & Safety Laboratory, Tucson, AZ, USA) via glass membrane feeders or were allowed to feed on CD-1 mice (Envigo, St. Louis, MO, USA) sedated with ketamine (50 mg/kg) and xylazine (5 mg/kg). Mouse protocols were performed at the University of Idaho and approved by the Animal Care and Use Committee and were in accordance with the federal regulatory guidelines and standards (University of Idaho IACUC-2020-10 protocol, approved 30 March 2020). For non-infectious experimental blood feeding, mosquitoes were provided with whole human blood (American Red Cross, Tucson, AZ, USA; IBC protocol 2010-014) via membrane feeders [12]. All adult female mosquitoes used in these studies were 3–7 d old.

### 2.3. Mosquito Dissections, RNA Isolation, cDNA Synthesis, and qPCR

Midguts of female *A. stephensi* were dissected prior to provisioning with human blood (non-blood-fed or NBF) and at 2, 6, 12, 24, 36, 48, 72 h post-blood feeding for *PanK* transcript analysis. Midgut dissections were performed in 1X PBS and stored in 25 μL RNAlater (Invitrogen, Waltham, MA, USA) at −80 °C. Total RNA was isolated from dissected midguts using the RNeasy mini kit (Qiagen, Germantown, MD, USA) and quantified using a Nanodrop 2000 Spectrophotometer (Thermo Scientific, Grand Island, NY, USA). Total RNA samples were treated with TURBO DNase I (Invitrogen, Waltham, MA, USA) as per the manufacturer’s protocol to eliminate genomic DNA contamination. The high-capacity cDNA reverse transcription kit (Applied Biosystems, Foster City, CA, USA) was used to synthesize cDNA using random hexamer primers according to the manufacturer’s protocol. Quantitative PCR was performed using the Maxima SYBR Green/ROX qPCR master mix (Thermo Scientific, Waltham, MA, USA) and *A. stephensi PanK* forward (5′ GGACAACTACAAGCGCATCTC 3′) and reverse (5′ TCACCCTTCGTAGCTAACTG 3′) primers.

### 2.4. A. stephensi PanK RNA Interference (RNAi)

PanK RNAi forward and reverse primers were designed using NetPrimer (PREMIER Biosoft, San Francisco, CA) to knock down *A. stephensi* PanK transcript: PanK RNAi-F 5′ TAATACGACTCACTATAGGGAGAACGCTGACGAAGCTGGTGTA 3′ and PanK RNAi-R 5′ TAATACGACTCACTATAGGGAGACGGTGAGCAGACAGCACAG 3′ (T7 RNA polymerase promoter sequence is underlined). The expected amplicon (607 bp) was PCR amplified using Taq 2X Master Mix (New England Biolabs, Ipswich, MA, USA) with *A. stephensi* midgut cDNA as the template. Double stranded RNA (dsRNA) was synthesized using HiScribe T7 Quick High Yield RNA Synthesis Kit (New England Biolabs, Ipswich, MA, USA). Cold-anesthetized female mosquitoes were intrathoracically microinjected twice with 276 nL dsRNA (8 μg/μL) using a Nanoject II microinjector (Drummond Scientific, Broomall, PA). The first injection was performed within 4 h of adult eclosion and the second injection was completed at 3 d post-eclosion. Injected mosquitoes were maintained on 10% sucrose throughout the experiments. Firefly luciferase dsRNA (dsRNA-FLuc) was synthesized and injected following the above protocol as a negative control [14,15]. Injected mosquitoes were provided a blood meal at 5 d after adult eclosion; dissected midguts were collected for analysis immediately prior to blood feeding (NBF) and at 2, 6, and 24 h post-blood feeding.

### 2.5. Coenzyme A Quantification

Coenzyme A (CoA) levels in midguts of female *A. stephensi* were measured using the CoA Assay Kit (Sigma-Aldrich, St. Louis, MO, USA). For this assay, midguts from 10 mosquitoes were dissected on ice in PBS and 1X protease inhibitor (Complete™ Mini Protease Inhibitor Cocktail; Sigma-Aldrich, St. Louis, MO, USA) from NBF mosquitoes and from mosquitoes at 2, 6, and 24 h post-blood feeding with the blood bolus removed. Midguts were transferred to 50 µL of ice-cold 5X protease inhibitor-PBS solution after which CoA assay buffer (50 μL) was added to the samples and the midguts were homogenized in 1.5 mL centrifuge tubes. CoA concentrations in two midgut equivalents (20 μL) from each homogenized sample were determined using an EPOCH/2 microplate reader (BioTek Instruments, Winooski, VT, USA) at 570 nm absorbance and a CoA standard curve as per the manufacturer’s instructions.

### 2.6. PanK Homology Modeling

Protein sequences for *A. stephensi* PanK and human PANK1, PANK2, and PANK3 were obtained from the Uniprot data base (https://www.uniprot.org/, accessed on 6 April 2021) and compared using Align Sequences Protein BLAST within the blastp suite [16]. Sequences were aligned using Clustal Omega (https://www.ebi.ac.uk/Tools/msa/clustalo/, accessed on 6 April 2021) and sequence conservation was visualized using ESPript (http://endscript.ibcp.fr/ESPript/ESPript/index.php, accessed on 6 April 2021). The *A. stephensi* PanK•ATP•Mg^2+^•PZ-2891 threaded structure was constructed using SWISS-MODEL (https://swissmodel.expasy.org/, accessed on 6 April 2021), Uniprot accession A0A182YPP9 and the template PANK3•ATP•Mg^2+^•PZ-2891 (PDB: 6B3V) [17]. The global model quality estimation was 0.8 and the QMEAN was −0.85 indicating high reliability, high expected accuracy, and good agreement between the model and the expected experimental structure. Coordinates were visualized using PyMOL.

### 2.7. Western Blotting

Midguts were dissected on ice from female *A. stephensi* prior to blood feeding (NBF) and 2, 6, 12, 24, 36, 48, and 72 h post-blood meal in PBS with 1X protease inhibitor and the blood bolus removed. Ten midguts were pooled and transferred to 25 µL of protease inhibitor (5X) solution with an equal volume of cell lysis buffer (1X PBS, 1% Triton X-100, 12 mM sodium deoxycholate, 2% SDS) and 25 µL of Laemmli sample buffer (50 mM Tris-HCl pH 6.8, 10% glycerol, 2% SDS, 0.2 mg/mL bromophenol blue, 0.1 M dithiothreitol). Lysed tissues were homogenized, denatured at 95 °C for 10 min, and centrifuged at 14,000× *g* for 5 min at room temperature. The supernatants were transferred to sterile microcentrifuge tubes and stored at −80 °C. For each protein sample, a single midgut equivalent was loaded onto precast 12% SDS-PAGE gels (NuSep, Germantown, MD, USA) with PageRuler™ Prestained Protein Ladder (Thermo Scientific, Waltham, MA, USA) as a molecular marker. Size-fractionated proteins were transferred to nitrocellulose membranes (LI-COR, Lincoln, NE, USA) for 1 h at 100 V. Membranes were blocked in non-fat milk (4% *w*/*v*) in 1X PBS (pH 7.4) for 1 h at room temperature, then incubated with 1:1000 *A. stephensi* PanK polyclonal antibody and 1:1000 alpha-tubulin monoclonal antibody (Developmental Studies Hybridoma Bank, Iowa City, IA, USA) in PBST (1X PBS with 0.1% Tween 20, Sigma, St. Louis, MO, USA) with non-fat milk (4% *w*/*v*) overnight at 4 °C. Following primary antibody incubation, membranes were washed ten times, 5 min each with 1X PBST, then incubated with secondary antibodies goat anti-rabbit 800 CW (1:10,000, LI-COR, Lincoln, NE, USA) and goat anti-mouse 680 RD (1:10,000, LI-COR, Lincoln, NE, USA) in PBST for 1 h at room temperature. The membranes were washed an additional ten times, 5 min each with PBST. A LI-COR imaging system with Image Studio software was used to acquire membrane signals. Densitometry quantification of protein bands was performed using ImageJ (NIH) as previously described [18].

### 2.8. P. falciparum NF54 In Vitro Growth Assay

Frozen stocks of *P. falciparum* NF54 infected RBCs were thawed at 37 °C, washed three times with NaCl (12%, 1.6% and 0.9% respectively) to remove glycerol and transferred to culture flasks [19]. Parasites were maintained at 37 °C in RPMI-1640 medium supplemented with HEPES, L-glutamine, hypoxanthine and DL-Lactic acid (supplemented RPMI) with 4.0-6.0% washed type O+ RBCs. Flask media were changed daily, followed by injection of mixed gas (5% CO_2_, 5% O_2_, 90% N_2_). Parasitemia was assessed via microscopic examination of thin films stained with Giemsa [20]. For growth assays, cultures were synchronized by the addition of sorbitol (5%, 1:25 *v*/*v*) and incubation at 37 °C for 5 min. The treated culture was centrifuged at 800× *g* for 10 min to pellet infected RBCs with young trophozoites, then resuspended in supplemented RPMI and returned to culture [21]. For growth assays, 200 μL of synchronized *P. falciparum* culture was transferred to each well of a 96-well plate at an initial parasitemia of 0.5–1.0% and hematocrit of 1.0% [22]. Growth was assessed at 48 h and 96 h or one and two parasite life cycles, respectively. Parasites were treated with chloroquine diphosphate (19.5, 39, 78 and 156 nM; Sigma) as a positive control for suppression of parasite growth. PZ-2891 effects on *P. falciparum* growth *in vitro* were tested at 1.3 nM (reported EC_50_ for human PanK3 [17]) and at 13 nM, 130 nM, and 1.3 µM, while compound 7 effects were tested at 62 nM (mean EC_50_ for human PanK3, PanK1β and PanK2 [23]) and at 620 nM, 6.2 µM and 62 µM. Control parasites were treated with a volume of DMSO equivalent to that added for PZ-2891 or compound 7. At 48 h or 96 h after treatment, samples were collected from the 96-well plate, fixed with 10% formalin (Sigma), stained with 10 µg/mL PI in PBS [13], and analyzed using flow cytometry (Beckman Coulter Life Sciences, Indianapolis, IN, USA) and CytExpert software for the CytoFLEX Platform.

### 2.9. P. falciparum NF54 Gametocyte Culture

*P. falciparum* NF54 culture was initiated at 6% hematocrit and 0.5% parasitemia, with most parasites at the immature trophozoite stage and maintained in supplemented RPMI [10] and gas mixture described above. At 5 d after culture initiation or when parasites are present as unhealthy young trophozoites, the hematocrit was reduced to 3–4% with the addition of medium. Stage IV and V gametocytes were typically observed 8–12 d following hematocrit reduction. The 14, 15, and 17 d old cultures were combined for mosquito feeding, where days are counted beginning with the culture dilution to 0.5% parasitemia at 6% hematocrit, after which only the medium was changed daily [24].

### 2.10. P. falciparum NF54 and P. yoelii yoelii 17XNL Infection of A. stephensi

*P. falciparum* gametocyte cultures were prepared as above for infection of adult female *A. stephensi*, but without synchronization. Exflagellation was confirmed on the day of mosquito feeding before addition of fresh media. About 10% sucrose-soaked cotton balls were removed from the mosquitoes 30 min to 1 h before feeding on a meal of 1:1 (*v*/*v*) human RBCs (35–45% infected RBCs, 55–65% uninfected RBCs) and heat-inactivated human serum, with the meal supplemented immediately before feeding with 1.3 nM or 13 nM PZ-2891, 62 nM or 620 nM compound 7 or an equivalent volume of DMSO used to deliver the treatments as a control. The *P. falciparum* infectious blood meal was provisioned via a Hemotek Insect Feeding System (Discovery Workshops, Accrington, UK) and glass bell feeders (Chemglass Life Sciences, Vineland, NJ, USA). Mosquitoes were allowed access to the blood meal for 15 min, after which partially fed and non-fed mosquitoes were removed from each group. Fed mosquitoes were returned to 10% sucrose-soaked cotton balls for nutrition and maintained accordingly until dissection to assess infection [19].

To test the effects of PZ-2891 and compound 7 on infection of *A. stephensi* by *P. y. yoelii* 17XNL, adult female mosquitoes were provisioned for 3 d prior to infection with 1.3 nM or 13 nM PZ-2891, 62 nM or 620 nM compound 7 or an equivalent volume of DMSO in water via soaked cotton balls changed twice daily. Female 8–10 week-old CD-1 mice (Envigo) were used for *P. y. yoelii* 17XNL infection of *A. stephensi*. The development and patterns of parasite infection in male and female CD-1 mice are identical, but female mice were used because they are larger and easier to manipulate. Mice were infected via intraperitoneal injection of 1 × 10^7^ *P. y. yoelii* 17XNL-infected RBCs, then monitored daily for parasitemia starting at 2 d post-infection (PI) via microscopic analysis of Giemsa-stained thin blood smears. Wet preps of blood drops were evaluated for exflagellation events per higher power field (HPF) of male gametocytes before mosquito feeding. Mice with similar exflagellation events per HPF were anesthetized and placed on mosquito cartons for 20 min to allow mosquitoes to feed. After mosquito feeding was complete, partially fed and non-fed mosquitoes were removed from each group and mice were euthanized by CO_2_ inhalation followed by cervical dislocation. Fed mosquitoes were maintained until dissection at 24 °C and 80% humidity with twice daily changes of PZ-2891, compound 7, or DMSO in water as soaked cotton balls. Mouse infection and euthanasia were conducted as approved by the Institutional Animal Care and Use Committee of the University of Idaho (IACUC-2020-10 protocol, approved 30 March 2020).

For both *P. falciparum* NF54 and *P. y. yoelii* 17XNL infection studies, midguts were dissected at 10 d PI and stained for 2 min in 1% mercurochrome for oocyst counting by microscopy. Infection studies with *P. falciparum* were completed with two separate biological cohorts of *A. stephensi* and two independent gametocyte cultures, while studies with *P. y. yoelii* 17XNL were completed with three separate biological cohorts of *A. stephensi* and three separate sets of infected mice. Sporozoite infections were analyzed in two of the three cohorts of *P. y. yoelii* 17XNL-infected *A. stephensi*. For these analyses, salivary glands were dissected at 12–15 d PI, with sporozoite infections scored on a scale of 1–4 per pair of glands, with 1 for 100–1000 sporozoites, 2 for 1000–10,000 sporozoites, 3 for 10,000–100,000 sporozoites, and 4 for 100,000+ sporozoites.

### 2.11. Statistical Analyses

*A. stephensi PanK* transcript and PanK protein levels, CoA concentrations, and *P. falciparum* NF54 in vitro growth data were analyzed using ANOVA and Tukey’s post hoc test or Student’s *t*-test. Infection intensity data (oocysts per midgut, salivary gland sporozoite scores) were analyzed using one-way Kruskal–Wallace ANOVA with Tukey’s post hoc test. Prevalences of *A. stephensi* infection were analyzed using Chi-square and Fisher’s exact tests. All differences were considered significant at α = 0.05.

## 3. Results

### 3.1. PanK Transcript and Protein Expression Were Induced in the A. stephensi Midgut Following Blood Feeding

In a previous study, we established that *A. stephensi PanK* transcript is expressed in the midgut following blood feeding [12]. Based on the midgut metabolomics data from the same study, we hypothesized that induction of mosquito *PanK* expression would be coincident with increased PanK protein and activity levels, which would shift available mosquito pantothenate to CoA. This could limit the amount of pantothenate available to the parasite and, thereby, reduce parasite infection of the mosquito host. To test the first part of our hypothesis, we sought to examine the transcript and protein expression of the *A. stephensi PanK* ortholog over time following a single blood meal using qPCR and Western blotting.

**Figure 1:**
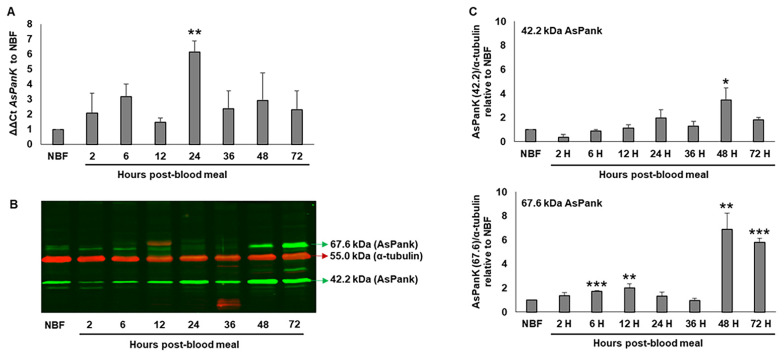
*A. stephensi PanK* transcript and protein levels in the midgut throughout a reproductive cycle. (**A**). *A. stephensi PanK* (*AsPanK*) transcript levels prior to (NBF) and following (2–72 h) a blood meal relative to a ribosomal S17 internal control. Error bars represent the standard error of the mean. Differences among respective timepoints were evaluated using one-way ANOVA with Tukey’s post hoc test. Three biological replicates with distinct cohorts of mosquitoes (ten mosquito midguts pooled per replicate) were performed. (**B**). Representative immunoblot showing changes in mosquito midgut PanK proteins prior to and during a reproductive cycle. α-tubulin antibody was used to assess loading. Three biological replicates with distinct cohorts of mosquitoes were performed and each lane represents one midgut equivalent from pools of ten mosquito midguts. (**C**). Densitometry analysis of the putative *A. stephensi* 42.2 and 67.6 kDa PanK isoforms from replicate immunoblots. Bars represent the density ratios of the respective PanK isoforms relative to α-tubulin. Error bars represent the standard error of the mean. Differences among respective timepoints compared to NBF were evaluated using one-way ANOVA with Tukey’s post hoc test. *p*-values: *** *p* < 0.001, ** *p* < 0.01, and * *p* < 0.05.

Following the blood meal, overall *A. stephensi PanK* transcript levels were significantly increased at 24 h post-blood meal, with a decline back to non-fed levels by 36 h (Figure 1A). In immunoblot assays of *A. stephensi* midgut proteins, we identified two distinct proteins that cross-reacted with *A. stephensi*-specific PanK antibody. The molecular weights of these two proteins (67.6 kDa and 42.2 kDa, green arrows, Figure 1B) were consistent with the predicted molecular weights of two putative splice variants identified in publicly available transcriptomic data (Vectorbase accession numbers ASTE005519 and ASTEI10435 [25]). Expression levels of the 42.2 kDa protein were significantly increased at 48 h post-blood feeding, whereas expression levels of the 67.6 kDa protein were increased early in the reproductive cycle (6 to 12 h post-blood meal) and by the end of egg development (48 and 72 h post-blood meal) relative to NBF controls (Figure 1C). Notably, the timing of induced *A. stephensi* PanK levels at 48 h post-blood meal would coincide with predicted ookinete to oocyst transition for *P. falciparum* [26] and early oocyst development for *P. y. yoelii* [27], suggesting that enhanced mosquito PanK activity from 48–72 h post-blood meal in an infected mosquito could result in competition between the mosquito host and rapidly growing parasite oocysts for available pantothenate.

### 3.2. Knockdown of A. stephensi PanK Significantly Reduced Midgut CoA Levels

**Figure 2:**
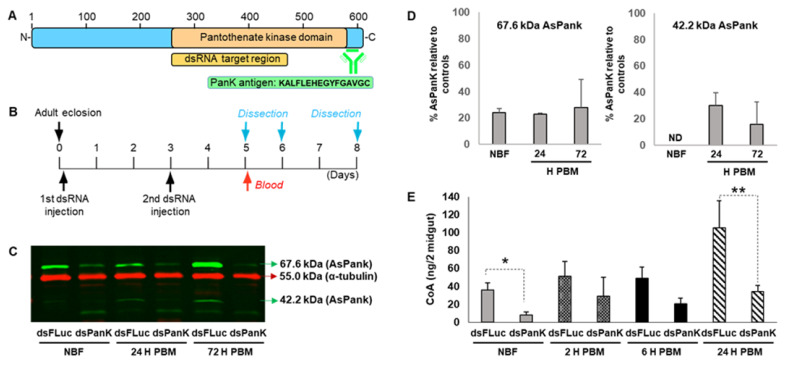
*A. stephensi PanK* RNAi reduced midgut PanK protein and coenzyme A (CoA) levels. (**A**). Schematic of *A. stephensi* PanK indicating the target location of the dsRNA construct and the peptide sequence used to generate the custom polyclonal antibody. (**B**). Experimental design for RNAi assays. *A. stephensi* females were injected with dsRNA targeting *A. stephensi* (*dsPanK*) or firefly luciferase (dsFLuc) within 4 h after adult eclosion and again at 3 d post-emergence. Mosquitoes were provided human blood on day 5 and midguts were dissected prior to blood feeding (non-blood-fed, NBF) and at 24 h and 72 h post-blood meal (PBM). (**C**). Representative immunoblot showing putative *A. stephensi* PanK isoforms (67.6 kDa and 42.2 kDa; green bands) following *PanK* RNAi relative to FLuc RNAi control (red band). Each lane represents one midgut equivalent from pools of ten mosquito midguts. RNAi and immunoblots were replicated twice with distinct cohorts of mosquitoes. (**D**). Densitometry analysis of the 67.6 kDa and 42.2 kDa *A. stephensi* PanK isoforms. Bars represent mean and standard deviation of percent PanK protein expression in mosquitoes inoculated with *dsPanK* relative to mosquitoes injected with dsFLuc. (**E**). Midgut CoA levels in dsPanK- and dsFLuc-treated *A. stephensi*. Differences between treatment groups for respective timepoints were evaluated using Student’s *t*-test. ** *p* < 0.01 and * *p* < 0.05. Experiments were replicated three times with independent cohorts of mosquitoes.

To confirm that *A. stephensi* PanK protein was functionally associated with CoA in vivo, we evaluated the effect of *PanK* RNAi on midgut *A. stephensi* CoA levels following a single blood meal (Figure 2A,B). Following *PanK* dsRNA treatment, we observed a significant reduction in the 67.6 kDa PanK isoform in both NBF (76% reduction) and blood-fed mosquitoes (77% reduction at 24 h and 72% reduction at 72 h) relative to FLuc controls (Figure 2C,D). The 42.2 kDa PanK isoform was expressed at low levels in the NBF RNAi-treated mosquitoes and thus knockdown was not determined (Figure 2C,D; right panel, ND). However, at 24 h and 72 h post-blood feeding, the 42.2 kDa PanK isoform was reduced 70% and 84%, respectively, relative to FLuc controls (Figure 2D). Importantly, *A. stephensi PanK* dsRNA treatment significantly reduced the midgut CoA levels in both NBF mosquitoes and in blood-fed mosquitoes at 24 h compared to FLuc controls (Figure 2E).

### 3.3. PanK Small Molecules Predictably Altered A. stephensi Midgut CoA Levels

While RNAi enabled us to confirm that *A. stephensi* PanK is functionally associated with midgut CoA levels, we sought to identify PanK-modulating small molecules that might ultimately be deployable to mosquitoes via attractive bait stations. To this end, we selected PZ-2891 and compound 7, which have been described as activating (EC_50_ of 1.3 nM for human PanK3 [17]) and inhibiting (mean EC_50_ of 62 nM for human PanK3, PanK1β and PanK2 [23]) human PanK catalytic activity, respectively, to test our hypothesis that activation of *A. stephensi* PanK would be associated with increased midgut CoA levels, while inhibition would be associated with reduced midgut CoA levels.

**Figure 3:**
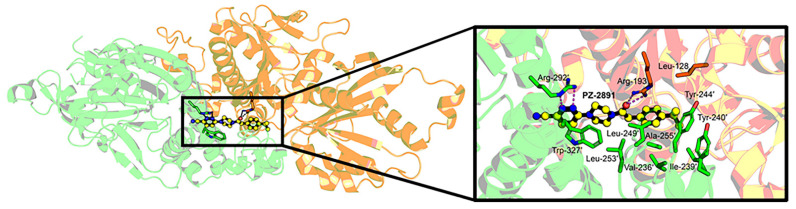
Homology model of *A. stephensi* PanK with bound PZ-2891. Interactions between PZ-2891 and *A. stephensi* PanK predict that the mosquito protein is regulated by the same allosteric mechanism described for PZ-2891 regulation of human PanKs. PZ-2891 interacts with both protomers through hydrophobic interactions, hydrogen bond contacts, and π-π stacking interactions. Sequence numbering is assigned to *A. stephensi* PanK. The two *A. stephensi* PanK protomers are colored green and orange and PZ-2891 is yellow.

The 3D structure of PZ-2891 has been published [17] and through homology modeling we compared the catalytic core of *A. stephensi* PanK (Uniprot: A0A182YPP9) to human PanK1 (Uniprot: Q8TE04-2), PanK2 (Uniprot: Q9BZ23), and PanK3 (Uniprot: Q9H999). *A. stephensi* PanK is 80% similar and 65% identical to human PanK3 and the conserved signature sequences [6]: xDIGGTLxKLxY, TGGGAxKF, VNxGSGVS, LGGGTFxGLCxLLT, DKLVxDIYGG, and GLxGxxVASSFG are present. The key catalytic residues Glu28 and Arg193 are essential for PanK function and distinguish bona fide PanKs from pseudo-PanKs (Supplementary Figure S1) [28]. This high sequence similarity supported the use of the PanK3 structure as a template for homology modeling. We used the coordinates of PanK3•ATP•Mg^2+^•PZ-2891 [17] to predict how *A. stephensi* PanK interacts with PZ-2891. The mosquito PanK has the identical residues that stabilize the allosteric dimer interface and interact with ATP, pantothenate, and acetyl-CoA in PanK3 (Figure 3) [29,30]. Residues surrounding the PZ-2891 molecule show the isopropyl moiety packs into a hydrophobic cavity of the pantothenate binding site, the carbonyl group hydrogen bonds to catalytic residue Arg193, and the pyridazine ring extends into the opposite protomer to form a hydrogen bond with Arg292′ and makes a p-p stacking interaction with Trp327′. These interactions are identical to the binding mode in PANK3 [17] and predict that *A. stephensi* PanK will be regulated by PZ-2891 by the same allosteric mechanism.

**Figure 4:**
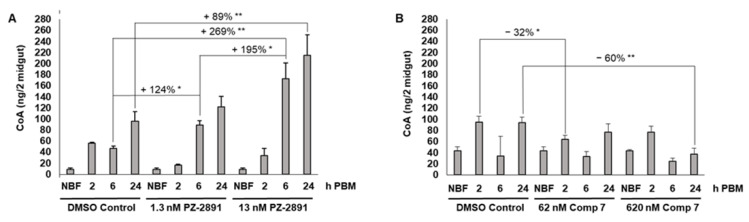
Oral provisioning of PZ-2891 increased and compound 7 decreased *A. stephensi* midgut CoA levels, respectively. (**A**)**.** Mosquitoes provisioned with PZ-2891 had significantly increased levels of midgut CoA at 6 h (13 nM and 1.3 nM) and 24 h (13 nM) relative to DMSO control, with a pattern of greater increases in midgut CoA for 13 nM vs. 1.3 nM PZ-2891 at 6 h and 24 h. Differences between treatments and controls were evaluated using Student’s *t*-test. ** *p* < 0.01 and * *p* < 0.05. Three distinct biological cohorts of mosquitoes were assayed. (**B**)**.** Mosquitoes provisioned with 620 nM and 62 nM compound 7 had reduced levels of midgut CoA at 24 h and 2 h, respectively, relative to DMSO control. Five distinct biological cohorts of mosquitoes were assayed. Differences between treatments and DMSO controls were evaluated using Student’s *t*-test. ** *p* < 0.01 and * *p* < 0.05. Five distinct biological cohorts of mosquitoes were assayed.

Based on these observations, we provisioned PZ-2891 (1.3 nM, 13 nM) and compound 7 (62 nM, 620 nM) to female *A. stephensi* in a meal of human blood delivered by artificial feeder. Both concentrations of PZ-2891 resulted in significantly increased midgut CoA levels at 6 h after the blood meal, with sustained effects of 13 nM PZ-2891 through 24 h and a pattern of greater increases in midgut CoA for 13 nM vs. 1.3 nM PZ-2891 at 6 h and 24 h (Figure 4A). In contrast, 620 nM compound 7 significantly reduced CoA levels at 24 h post-feeding relative to control, while 62 nM significantly reduced CoA at 2 h post-feeding relative to control (Figure 4B).

### 3.4. PZ-2891 and Compound 7 at Doses Used in Our Biological Assays Had No Direct Effects on P. falciparum NF54 Growth In Vitro

**Figure 5:**
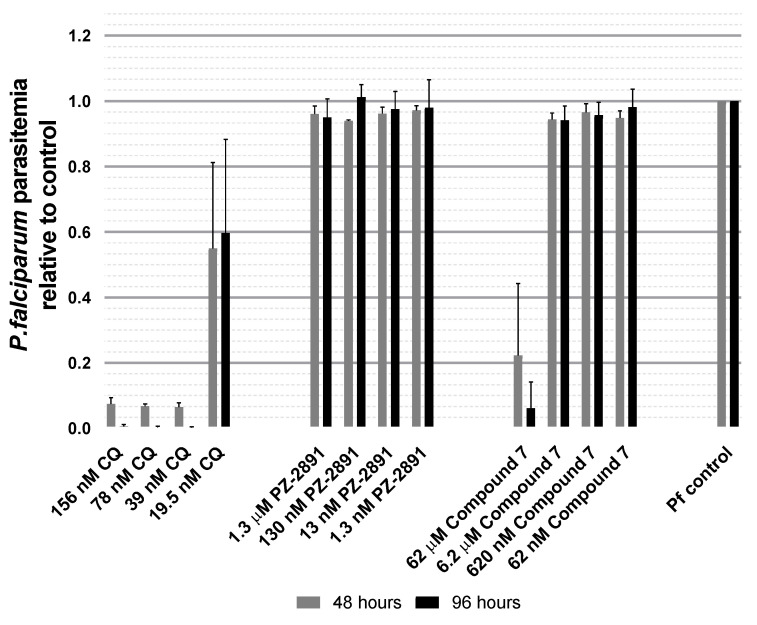
PZ-2891 and compound 7 at doses used in our biological assays had no direct effects on *P. falciparum* NF54 growth in vitro. Synchronized asexual stage *P. falciparum* NF54 strain was grown and treated with medium supplemented with chloroquine as a positive control for parasite killing (156 nM, 78 nM, 39 nM or 19.5 nM), PZ-2891 (1.3 µM, 130 nM, 13 nM or 1.3 nM), compound 7 (62 µM, 6.2 µM, 620 nM or 62 nM) or DMSO as a negative control at a volume equivalent to that used to deliver small molecules (indicated as “Pf control”, set to 1). Samples were collected 48 h and 96 h after treatment, stained with PI and analyzed by flow cytometry. The data are represented as mean parasitemia +/− SEM of the treated cultures relative to Pf control. The assays were replicated twice with distinct parasite cultures.

Both *A. stephensi* and *Plasmodium* spp. utilize pantothenate to synthesize CoA. Our long-term goal is to specifically activate *A. stephensi* PanK to shift CoA biosynthesis in favor of the mosquito host to reduce available pantothenate for parasite infection and sexual stage development. To explore this hypothesis, we first sought to test PZ-2891 and compound 7 for effects on parasite growth in vitro, a proxy assay that would help to establish whether these compounds have any direct negative effects on parasite growth. To this end, we tested both PZ-2891 and compound 7 against growth of cultured *P. falciparum* NF54 in vitro. Growth of *P. falciparum* in replicate assays was quantified after 48 h (one full life cycle) and 96 h (two cycles) in groups treated with chloroquine as a positive control for growth inhibition (19.5–156 nM), with PZ-2891 (1.3 Nm–1.3 μM), with compound 7 (62 nM–62 μM), or with a volume of DMSO equivalent to the volume added for the small molecules as a diluent control (normalized to 1 and labeled as “Pf control”, Figure 5). In both replicates, chloroquine treatment reduced *P. falciparum* growth relative to control. However, none of the PZ-2891 doses, and only the highest compound 7 dose (62 μM, 100-fold higher than required for significant reduction in midgut CoA levels, Figure 4), had any effect on parasite growth relative to control (Figure 5). While these data do not confirm that PZ-2891 and compound 7 are completely inactive against mosquito-stage parasites, there is no feasible, high throughput assay to test compound activity against similar quantities of mosquito-stage parasites. Accordingly, we inferred that oral provisioning of compound 7 ≤ 620 nM and PZ-2891 ≤ 13 nM to *A. stephensi* would only indirectly impact the parasite survival through effects on mosquito host CoA biosynthesis.

### 3.5. PZ-2891 Decreased and Compound 7 Increased Infection Success of A. stephensi with Two Biologically Distinct Plasmodium Species

**Figure 6:**
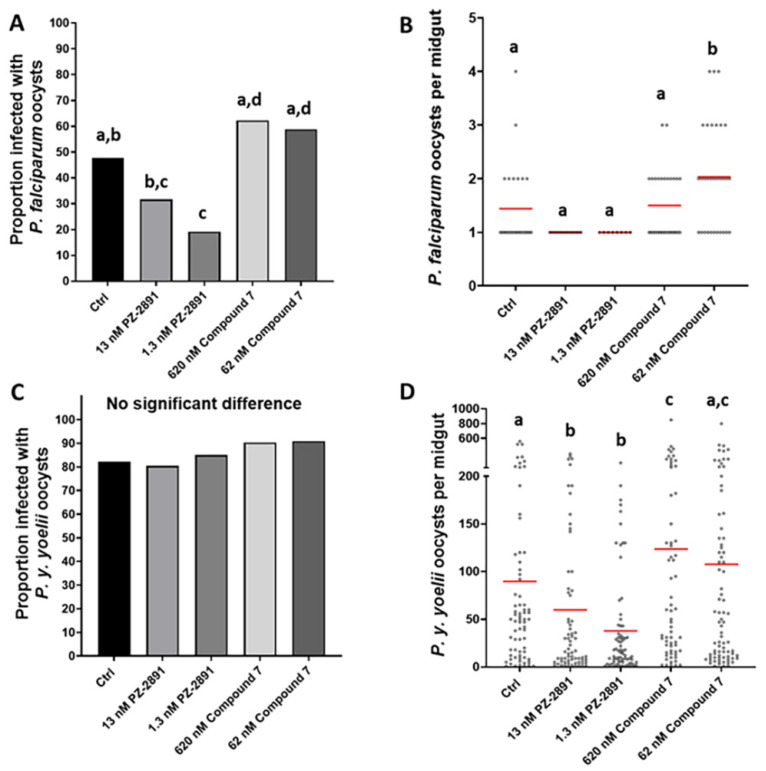
PZ-2891 and compound 7 decreased and increased, respectively, *P. y. yoelii* 17XNL and *P. falciparum* infection of *A. stephensi*. Graphs A and C represent infection prevalence for *P. yoelii* and *P. falciparum,* respectively, while B and D show infection intensity. Prevalence data (A,C) were analyzed by Chi-square and Fisher’s exact tests and infection intensity data (B,D) were analyzed by one-way ANOVA and Tukey’s post hoc test. Different lower case letters above data columns indicate groups that are significantly different by Chi-square (graphs A,C) or ANOVA and Tukey’s (graphs B,D) at the α = 0.05 level of significance (i.e., data indicated as “a” are significantly different from data indicated as “b” within a single graph). (**A**). *P. falciparum* infection prevalence. Specific *p*-values for significant differences are as follows: Control (Ctrl) vs. 1.3 nM PZ-2891 *p* = 0.0073; 13 nM PZ-2891 vs. 620 nM compound 7 *p* = 0.0009; 13 nM PZ-2891 vs. 62 nM compound 7 *p* = 0.0022; 1.3 nM PZ-2891 vs. 620 nM compound 7 *p* < 0.0001; 1.3 nM PZ-2891 vs. 62 nM compound 7 *p* = 0.0001. Two biological replicates were performed with unique cohorts of mosquitoes (n = 28–30 mosquitoes per control and treatment groups). (**B**). *P. falciparum* infection intensity. Numbers of oocysts per midgut of individual female *A. stephensi* mosquitoes infected with *P. falciparum* are represented by black circles. Red bars represent mean oocysts/midgut. Specific *p*-values for significant differences are as follows: Ctrl vs. 62 nM compound 7 *p* = 0.0266; 13 nM PZ-2891 vs. 62 nM compound 7 *p* = 0.0004; 1.3 nM PZ-2891 vs. 62 nM compound 7 *p* = 0.0051; 620 nM compound 7 vs. 62 nM compound 7 *p* = 0.0424. (**C**). *P. yoelii* prevalence. No significant differences were observed among groups, likely due to the high infection prevalence overall. Three biological replicates were performed with unique cohorts of mosquitoes (n = 70–88 mosquitoes per group). (**D**). *P. yoelii* infection intensity. Numbers of oocysts per midgut of female *A. stephensi* mosquitoes infected with *P. yoelii.* Red bars represent mean oocysts/midgut. Specific *p*-values for significant differences are as follows: Ctrl vs. 13 nM PZ-2891 *p* = 0.0164; Ctrl vs. 1.3 nM PZ-2891 *p* = 0.0003; Ctrl vs. 620 nM compound 7 *p* = 0.055; 13 nM PZ-2891 vs. 620 nM compound 7 *p* = 0.0009; 13 nM PZ-2891 vs. 62 nM compound 7 *p* = 0.0081; 1.3 nM PZ-2891 vs. 620 nM compound 7 *p* < 0.0001; 1.3 nM PZ-2891 vs. 62 nM compound 7 *p* < 0.0001.

To assess the impact of PZ-2891 and compound 7 on parasite infection in *A. stephensi*, female mosquitoes were provisioned with these compounds via soaked cotton balls prior to and following the exposure to mice infected with *P. y. yoelii* 17XNL or via a *P. falciparum*-infected blood meal supplemented with these compounds immediately before feeding. As expected based on the effects on midgut CoA levels, 1.3 nM PZ-2891 delivered one time in the infected blood meal reduced the proportion of *A. stephensi* infected with *P. falciparum*, with a trend toward reduced infection with 13 nM PZ-2891, while treatment with compound 7 significantly increased the proportion of infected mosquitoes relative to PZ-2891 treatment but not relative to control (Figure 6A). Mean *P. falciparum* oocysts per midgut (range 1–6 oocysts) were significantly increased by 62 nM compound 7 (Figure 6B). It is important to note, however, that a single oocyst can produce enough sporozoites for transmission, so increasing the proportion of mosquitoes that are not infected (i.e., reducing prevalence) is necessary to reduce transmission. In contrast to the results with *P. falciparum*, pre- and post-infection treatment with PZ-2891 and compound 7 had no effect on the proportions of *A. stephensi* infected with *P. y. yoelii* 17 XNL oocysts and sporozoites (Figure 6C, Supplementary Figure S2), likely because overall infection levels were very high. Despite these high infection levels, treatment with both concentrations of PZ-2891 and 620 nM compound 7 significantly reduced and increased, respectively, mean *P. y. yoelii* 17XNL oocysts per midgut relative to control (Figure 6D). While such infection levels with this mouse parasite are far from natural and these reductions would have no impact on sporozoite transmission, these results suggest that these small molecules can alter a very large parasite biomass when delivered daily. Despite the differences in the magnitudes and patterns of these effects, PZ-2891 significantly reduced infection success of both *P. falciparum* and *P. y. yoelii* 17XNL in *A. stephensi*, two parasite species that have markedly different genetics and life cycle biology [31].

## 4. Discussion

The absolute requirement for exogenous pantothenate by *Plasmodium* spp. makes it an attractive target for parasite control in both the vertebrate host and mosquito vector [2]. Depletion of pantothenate in the mosquito through manipulation of CoA biosynthesis is expected to negatively impact *Plasmodium* survival by starving the parasite of this essential nutrient. PanK is a logical target to assess whether pantothenate depletion in the mosquito can impact parasite development as it is the first enzyme in the CoA biosynthesis pathway. PanK is highly conserved across a range of organisms, and while there is limited research on PanK biology in invertebrates, putative *PanK* orthologs have been identified from numerous arthropod genomes. *Drosophila melanogaster* encodes a single *PanK* gene *fumble* (*fbl*), which is the source of seven transcript variants (*fbl-RA* to *fbl-RG*) [32,33]. Flies with *fbl* mutations have a shortened adult lifespan, sterility, neurodegeneration and typically perish before adult eclosion [32,34]. We identified at least two putative *A. stephensi* PanK isoforms with predicted molecular weights of 67.6 and 42.2 kDa, which correspond to the major protein bands that cross-reacted with our *A. stephensi* PanK antibody (Figure 1). Despite variable PanK N-termini, the C-terminal kinase domains of *D. melanogaster* PanK and the predicted *A. stephensi* PanK proteins are highly conserved, suggesting that core kinase activity is conserved across these variants.

Our RNAi data confirmed that the 67.6 kDa and 42.2 kDa proteins are *A. stephensi* PanK isoforms based on the robust reduction of both protein bands following the inoculation of *PanK* dsRNA but not *Fluc* dsRNA. Furthermore, the lower molecular weight protein (~30 kDa) we routinely observed appears to be a non-specific target of the PanK antibody, since treatment with *PanK* dsRNA had no effect on levels of this protein relative to the FLuc control (Figure 2C). In the *A. stephensi* midgut, levels of both PanK proteins were present at fairly constant levels prior to and throughout most of reproductive cycle (Figure 1). However, for the 67.6 kDa protein, and to a lesser extent the 42.2 kDa protein, we observed an increase in protein expression at the end of the reproductive cycle following vitellogenesis and after oviposition. This may reflect an effort by the mosquito to replenish stores of free fatty acids following the provisioning of triglycerides into the developing oocytes [35]. Increased PanK levels would facilitate the conversion of pantothenate to CoA and subsequently to acetyl-CoA for the synthesis of fatty acids [36,37]. A significant increase in *A. stephensi PanK* transcript observed at 24 h post-blood meal in the midgut likely drives the increase in the 42.2 kDa protein at 24 h post-blood meal and an overall increase in PanK protein levels starting by 48 h post-blood meal.

We also utilized RNAi to verify the core biological function of *A. stephensi* PanK, which is to initiate the catalytic conversion of pantothenate to CoA. The significant reduction in midgut CoA levels following RNAi treatment prior to blood feeding and at 24 h post-blood feeding confirmed this to be the case. Interestingly, we did not observe a significant decrease in midgut CoA shortly after blood feeding. This could be due to a combination of modest PanK protein levels at this time point, biological variation among mosquito cohorts, unknown regulators of the CoA biosynthesis pathway in mosquitoes, and the complex events occurring during the initial stages of blood meal digestion. While *A. stephensi PanK* RNAi was effective and the outcome consistent with the reported biological function of PanK in other organisms, it would be difficult to translate genetic manipulation to a feasible anti-*Plasmodium* control strategy. Thus, we tested the utility of orally available small molecules to manipulate *A. stephensi* PanK signaling and associated biology.

We tested two well-characterized small molecules for modulation of *A. stephensi* PanK activity. At modest concentrations, PZ-2891 interacts with one of the binding pockets of the human PanK3 dimer, which in turn locks the dimer into an active state and confers resistance to inhibition by acetyl-CoA, allowing it to function as a PanK activator [17]. At higher concentrations, PZ-2891 may interact with both pockets of the PanK dimer, interfering with its activity. Although we did not directly verify the binding of PZ-2891 to recombinant *A. stephensi* PanK due to the substantial technical challenges involved with such work, the high degree of sequence conservation between human PanK3 and *A. stephensi* PanK and the threaded model of *A. stephensi* PanK suggests that PZ-2891 would have similar biological effects in *A. stephensi* (Figure 3, Supplementary Figure S1). The kinetic characteristics and the potential for acetyl-CoA inhibition of *A. stephensi* PanK should, however, be confirmed to support this. Treatment with PZ-2891 at both 1.3 nM and 13 nM was associated with significantly increased midgut CoA levels (Figure 4A), suggesting that this compound can act as a PanK activator over at least a ten-fold concentration range. Compound 7 has been shown to suppress human PanK3 activity and provisioning of 62 and 620 nM compound 7 to *A. stephensi* reduced midgut CoA levels at 2 h and 24 h post-blood meal, respectively (Figure 4B).

Based on the predicted biological effects of these two small molecules on midgut CoA levels in the mosquito, we examined the effects of these compounds on *Plasmodium* spp. infection in *A. stephensi*. Since pantothenate is essential for parasite development and cannot be synthesized de novo by *Plasmodium* spp., we anticipated that these compounds would alter infection with diverse parasite species [3]. A growth assay of cultured asexual *P. falciparum* allowed us to verify that neither PZ-2891 nor compound 7 were directly toxic to these parasite stages, with the exception of 62 μM compound 7, which was 100 times the maximum concentration used in our infection assays. While these data do not rule out all possible effects of the small molecules on the parasite itself, they do suggest that these molecules do not interact effectively with the *P. falciparum* PanK. This would be expected since *P. falciparum* PanK proteins share less than 30% sequence identity with *A. stephensi* PanK. As discussed above, the *P. yoelii* genome encodes two PanK orthologs, neither of which share strong sequence identify to *A. stephensi* PanK [6]. Furthermore, the recent discovery that *P. falciparum* PanK1 and PanK2 are complexed with a dimer of sporozoite-specific 14-3-3I suggests that while asexual stage parasite PanK has a much higher affinity for pantothenate than mammalian PanK [38], activity of parasite PanK in the mosquito host may be substantially altered by its interaction with 14-3-3I [39]. Notably, a large array of animal and plant 14-3-3 proteins alters the cellular localization of their bound proteins, the interaction of bound proteins with other proteins, as well as the biological activity of bound proteins [40]. Accordingly, the upregulation of sporozoite-specific 14-3-3I in both oocyst-stage and salivary gland sporozoites of both *P. falciparum* and *P. y. yoelii* 17XNL could alters the biochemical activity of parasite PanK during these stages [41], perhaps making the mosquito host a more amenable target for blocking parasite utilization of host pantothenate.

The long-term goal of this work is to develop a bait-station approach to deliver small molecules to mosquitoes to divert pantothenate stores into CoA, effectively depriving developing malaria parasites of this essential molecule. As described above, provisioning with PZ-2891 at both 1.3 and 13 nM significantly reduced infection with both *Plasmodium* spp., demonstrating that manipulation of the *A. stephensi* CoA biosynthesis pathway can effectively limit parasite development. Importantly, we believe this approach has the potential to be effective across a range of mosquito and *Plasmodium* parasite combinations for several reasons. First, this approach is based on limiting pantothenate availability to the parasite, rather than directly targeting gene products in the parasite that may vary across species. This is supported by our results demonstrating the ability of PZ-2891 to suppress mosquito infection with two biologically distinct *Plasmodium* species. Furthermore, by not directly targeting the parasite for killing, we reduce the likelihood of the parasite developing resistance against this approach [42]. Second, the highly conserved nature of the CoA biosynthesis pathway across anopheline species suggests that optimized small molecule PanK regulators should behave similarly across a range of mosquito vectors (e.g., 99% amino acid identity between the catalytic domains of *A. stephensi* and *A. gambiae* PanK). Third, driving pantothenate through the CoA pathway in the mosquito, while deleterious to the *Plasmodium* parasite, is unlikely to impact the fitness of adult mosquitoes since the overall availability of CoA to the mosquito host would not be affected. Nevertheless, future studies on the impact of provisioning optimized mosquito-targeted small molecules on mosquito fitness are needed prior to transitioning this approach to field studies.

In this work we demonstrated that PanK biology and its regulation of CoA biosynthesis is conserved in an important and highly invasive malaria vector mosquito. As in other organisms, multiple isoforms of PanK were predicted and at least two were confirmed to be expressed in the *A. stephensi* midgut. Most importantly, we demonstrated that manipulation of *A. stephensi* PanK activity can significantly impact the development of multiple *Plasmodium* species in the insect host. As demonstrated by Schalkwijk et al. [9], pantothenamides can be used to effectively kill parasites in the vertebrate host by interfering with *Plasmodium* utilization of CoA. However, the risk of parasites developing resistance to these small molecules is a concern, as demonstrated by the ability to select for pantothenamide-resistant parasites [9]. Our results suggest that small molecules capable of modifying CoA biosynthesis activity in the mosquito could be used in a multifaceted control strategy to limit *Plasmodium* development in the mosquito vector, while simultaneously preserving the efficacy of pantothenamides as a novel therapeutic strategy in humans.

## Data Availability

The data presented in this study are available on request from the corresponding author.

## Supplementary Materials

The following are available online at https://www.mdpi.com/article/10.3390/biom11060807/s1. Supplemental Figure S1: Conserved features in eukaryotic pantothenate kinases. Sequence alignment of the *A. stephensi* PanK (AsPanK) catalytic core with human PanK1, PanK2, and PanK3 illustrates six conserved signature sequences [6]: xDIGGTLxKLxY, TGGGAxKF, VNxGSGVS, LGGGTFxGLCxLLT, DKLVxDIYGG, and GLxGxxVASSFG. Conserved catalytic residues Glu-128 and Arg-193 are essential for PanK function [28] and present in *A. stephensi* PanK denoted by blue arrows. Sequence numbering is assigned to *A. stephensi* PanK. Supplemental Figure S2: Oral provisioning of PZ-2891 and compound 7 had no effects on *P. y. yoelii* 17XNL sporozoite infection of *A. stephensi*. Sporozoite infections were analyzed in two of the three separate cohorts of *P. y. yoelii* 17XNL-infected *A. stephensi* from Figure 6. For these analyses, salivary glands were dissected at 12–15 d PI, with sporozoite infections scored on a scale of 1–4 per pair of glands, with 1 for 100–1000 sporozoites, 2 for 1000–10,000 sporozoites, 3 for 10,000–100,000 sporozoites, and 4 for 100,000+ sporozoites. Prevalence data or proportions of mosquitoes with sporozoite-positive salivary glands (A) were analyzed by Chi-square and Fisher’s exact tests and infection intensity data (B) were analyzed by one-way ANOVA.

## References

[1] Spry C., Van Schalkwyk D., Strauss E., Saliba K. (2010). Pantothenate utilization by Plasmodium as a target for antimalarial chemotherapy. Infect. Disord.-Drug Targets (Former. Curr. Drug Targets-Infect. Disord.).

[2] Hart R.J., Abraham A., Aly A.S. (2017). Genetic characterization of coenzyme A biosynthesis reveals essential distinctive functions during malaria parasite development in blood and mosquito. Front. Cell. Infect. Microbiol..

[3] Rock C.O., Calder R.B., Karim M.A., Jackowski S. (2000). Pantothenate kinase regulation of the intracellular concentration of coenzyme A. J. Biol. Chem..

[4] Sinka M., Pironon S., Massey N., Longbottom J., Hemingway J., Moyes C., Willis K. (2020). A new malaria vector in Africa: Predicting the expansion range of Anopheles stephensi and identifying the urban populations at risk. Proc. Natl. Acad. Sci. USA.

[5] Zhyvoloup A., Nemazanyy I., Babich A., Panasyuk G., Pobigailo N., Vudmaska M., Naidenov V., Kukharenko O., Palchevskii S., Savinska L. (2002). Molecular cloning of CoA synthase: The missing link in CoA biosynthesis. J. Biol. Chem..

[6] Hart R.J., Cornillot E., Abraham A., Molina E., Nation C.S., Mamoun C.B., Aly A.S. (2016). Genetic characterization of Plasmodium putative pantothenate kinase genes reveals their essential role in malaria parasite transmission to the mosquito. Sci. Rep..

[7] Tjhin E.T., Howieson V.M., Spry C., van Dooren G.G., Saliba K.J. (2021). A novel heteromeric pantothenate kinase complex in apicomplexan parasites. bioRxiv.

[8] Tjhin E.T., Spry C., Sewell A.L., Hoegl A., Barnard L., Sexton A.E., Siddiqui G., Howieson V.M., Maier A.G., Creek D.J. (2018). Mutations in the pantothenate kinase of Plasmodium falciparum confer diverse sensitivity profiles to antiplasmodial pantothenate analogues. PLoS Pathog..

[9] Schalkwijk J., Allman E.L., Jansen P.A., De Vries L.E., Verhoef J.M., Jackowski S., Botman P.N., Beuckens-Schortinghuis C.A., Koolen K.M., Bolscher J.M. (2019). Antimalarial pantothenamide metabolites target acetyl–coenzyme A biosynthesis in Plasmodium falciparum. Sci. Transl. Med..

[10] de Villiers M., Macuamule C., Spry C., Hyun Y.-M., Strauss E., Saliba K.J. (2013). Structural modification of pantothenamides counteracts degradation by pantetheinase and improves antiplasmodial activity. ACS Med. Chem. Lett..

[11] Spry C., Chai C.L., Kirk K., Saliba K.J. (2005). A class of pantothenic acid analogs inhibits Plasmodium falciparum pantothenate kinase and represses the proliferation of malaria parasites. Antimicrob. Agents Chemother..

[12] Souvannaseng L., Hun L.V., Baker H., Klyver J.M., Wang B., Pakpour N., Bridgewater J.M., Napoli E., Giulivi C., Riehle M.A. (2018). Inhibition of JNK signaling in the Asian malaria vector Anopheles stephensi extends mosquito longevity and improves resistance to Plasmodium falciparum infection. PLoS Pathog..

[13] Baniecki M.L., Wirth D.F., Clardy J. (2007). High-throughput Plasmodium falciparum growth assay for malaria drug discovery. Antimicrob. Agents Chemother..

[14] Isoe J., Collins J., Badgandi H., Day W.A., Miesfeld R.L. (2011). Defects in coatomer protein I (COPI) transport cause blood feeding-induced mortality in Yellow Fever mosquitoes. Proc. Natl. Acad. Sci. USA.

[15] Scaraffia P.Y., Tan G., Isoe J., Wysocki V.H., Wells M.A., Miesfeld R.L. (2008). Discovery of an alternate metabolic pathway for urea synthesis in adult Aedes aegypti mosquitoes. Proc. Natl. Acad. Sci. USA.

[16] Altschul S.F., Madden T.L., Schäffer A.A., Zhang J., Zhang Z., Miller W., Lipman D.J. (1997). Gapped BLAST and PSI-BLAST: A new generation of protein database search programs. Nucleic Acids Res..

[17] Sharma L.K., Subramanian C., Yun M.-K., Frank M.W., White S.W., Rock C.O., Lee R.E., Jackowski S. (2018). A therapeutic approach to pantothenate kinase associated neurodegeneration. Nat. Commun..

[18] Davarinejad H. Quantifications of Western Blots with ImageJ. www.yorku.ca/yisheng/Internal/Protocols/ImageJ.pdf.

[19] Ribacke U., Moll K., Albrecht L., Ismail H.A., Normark J., Flaberg E., Szekely L., Hultenby K., Persson K.E., Egwang T.G. (2013). Improved in vitro culture of Plasmodium falciparum permits establishment of clinical isolates with preserved multiplication, invasion and rosetting phenotypes. PLoS ONE.

[20] West R., Sullivan D.J. (2020). Lactic acid supplementation increases quantity and quality of gametocytes in Plasmodium falciparum culture. Infect. Immun..

[21] Lambros C., Vanderberg J.P. (1979). Synchronization of Plasmodium falciparum erythrocytic stages in culture. J. Parasitol..

[22] Desjardins R.E., Canfield C., Haynes J., Chulay J. (1979). Quantitative assessment of antimalarial activity in vitro by a semiautomated microdilution technique. Antimicrob. Agents Chemother..

[23] Sharma L.K., Leonardi R., Lin W., Boyd V.A., Goktug A., Shelat A.A., Chen T., Jackowski S., Rock C.O. (2015). A high-throughput screen reveals new small-molecule activators and inhibitors of pantothenate kinases. J. Med. Chem..

[24] Carter R., Ranford-Cartwright L., Alano P. (1993). The culture and preparation of gametocytes of Plasmodium falciparum for immunochemical, molecular, and mosquito infectivity studies. Protocols in Molecular Parasitology.

[25] Giraldo-Calderón G.I., Emrich S.J., MacCallum R.M., Maslen G., Dialynas E., Topalis P., Ho N., Gesing S., Consortium V., Madey G. (2015). VectorBase: An updated bioinformatics resource for invertebrate vectors and other organisms related with human diseases. Nucleic Acids Res..

[26] Meis J., Ponnudurai T. (1987). Ultrastructural studies on the interaction of Plasmodium falciparum ookinetes with the midgut epithelium of Anopheles stephensi mosquitoes. Parasitol. Res..

[27] Vaughan J.A., Hensley L., Beier J.C. (1994). Sporogonic development of Plasmodium yoelii in five anopheline species. J. Parasitol..

[28] Yao J., Subramanian C., Rock C.O., Jackowski S. (2019). Human pantothenate kinase 4 is a pseudo—Pantothenate kinase. Protein Sci..

[29] Hong B.S., Senisterra G., Rabeh W.M., Vedadi M., Leonardi R., Zhang Y.-M., Rock C.O., Jackowski S., Park H.-W. (2007). Crystal structures of human pantothenate kinases: Insights into allosteric regulation and mutations linked to a neurodegeneration disorder. J. Biol. Chem..

[30] Subramanian C., Yun M.-K., Yao J., Sharma L.K., Lee R.E., White S.W., Jackowski S., Rock C.O. (2016). Allosteric regulation of mammalian pantothenate kinase. J. Biol. Chem..

[31] Carlton J.M., Angiuoli S.V., Suh B.B., Kooij T.W., Pertea M., Silva J.C., Ermolaeva M.D., Allen J.E., Selengut J.D., Koo H.L. (2002). Genome sequence and comparative analysis of the model rodent malaria parasite Plasmodium yoelii yoelii. Nature.

[32] Afshar K., Gönczy P., DiNardo S., Wasserman S.A. (2001). fumble encodes a pantothenate kinase homolog required for proper mitosis and meiosis in Drosophila melanogaster. Genetics.

[33] Wu Z., Li C., Lv S., Zhou B. (2009). Pantothenate kinase-associated neurodegeneration: Insights from a Drosophila model. Hum. Mol. Genet..

[34] Yang Y., Wu Z., Kuo Y., Zhou B. (2005). Dietary rescue of fumble—a Drosophila model for pantothenate-kinase-associated neurodegeneration. J. Inherit. Metab. Dis..

[35] Van Antwerpen R., Pham D.Q.-D., Ziegler R. (2005). Accumulation of lipids in insect oocytes. Reproductive Biology of Invertebrates.

[36] Van Handel E. (1965). The obese mosquito. J. Physiol..

[37] Wakil S.J., Stoops J.K., Joshi V.C. (1983). Fatty acid synthesis and its regulation. Annu. Rev. Biochem..

[38] Saliba K.J., Kirk K. (2001). H+-coupled pantothenate transport in the intracellular malaria parasite. J. Biol. Chem..

[39] Meerstein-Kessel L., Venhuizen J., Garza D., Vos E.J., Obiero J.M., Felgner P.L., Sauerwein R.W., Peters M., Yang A.S., Huynen M.A. (2020). Novel functional insights from the Plasmodium falciparum sporozoite-specific proteome by probabilistic integration of 26 studies. bioRxiv.

[40] Yaffe M.B. (2002). How do 14-3-3 proteins work?—Gatekeeper phosphorylation and the molecular anvil hypothesis. FEBS Lett..

[41] Lindner S.E., Swearingen K.E., Shears M.J., Walker M.P., Vrana E.N., Hart K.J., Minns A.M., Sinnis P., Moritz R.L., Kappe S.H. (2019). Transcriptomics and proteomics reveal two waves of translational repression during the maturation of malaria parasite sporozoites. Nat. Commun..

[42] Glennon E.K., Dankwa S., Smith J.D., Kaushansky A. (2018). Opportunities for host-targeted therapies for malaria. Trends Parasitol..

