# Supplementary figures and images for: Activation of Anopheles stephensi Pantothenate Kinase and Coenzyme A Biosynthesis Reduces Infection with Diverse Plasmodium Species in the Mosquito Host
Source: Biomolecules. 2021 May 29;11(6):807. doi: 10.3390/biom11060807 (PMC8228300; doi:10.3390/biom11060807)

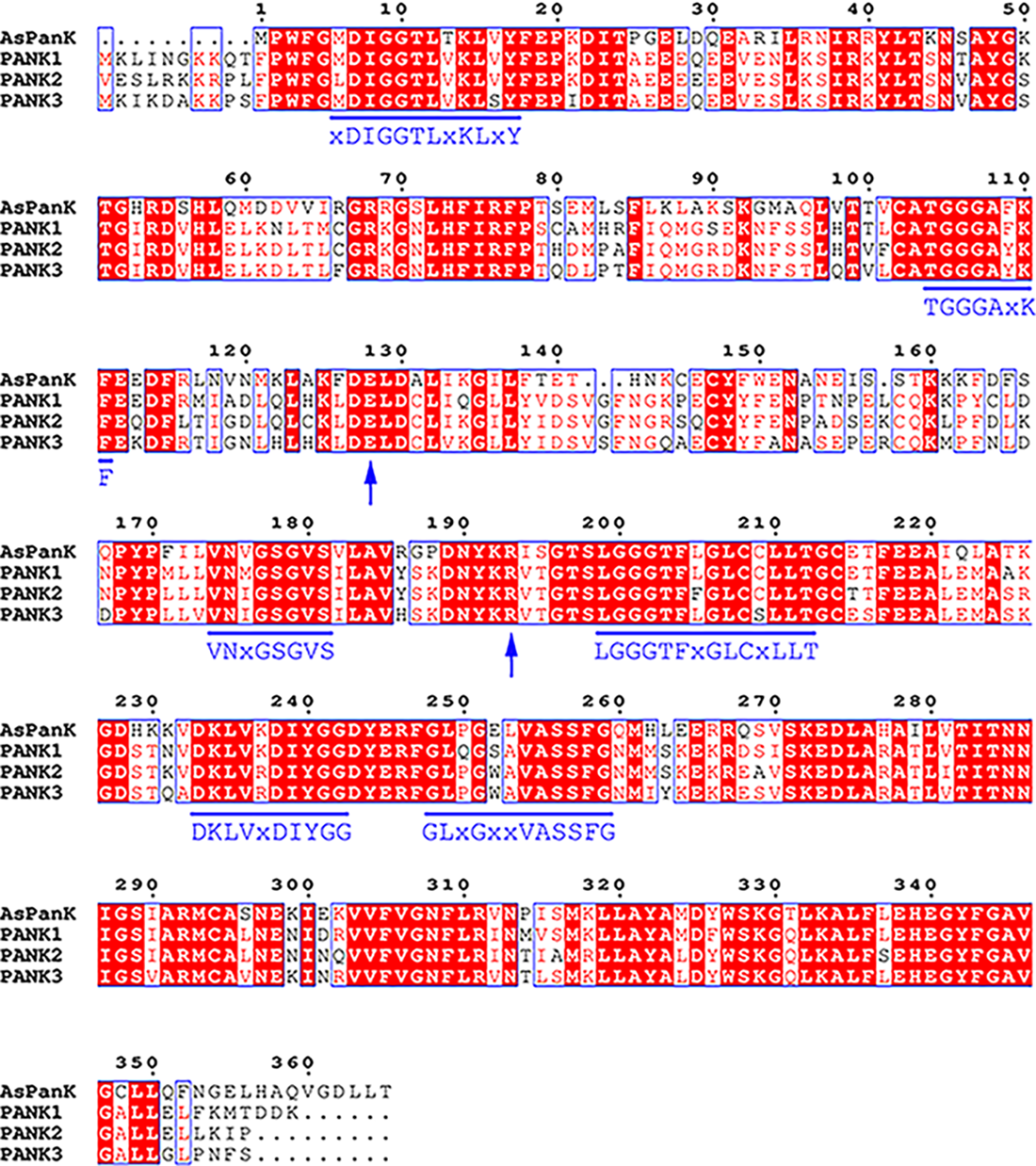

Supplement: Supplementary file 1 [file biomolecules-11-00807-s001.zip › Suppl Figure 1.tif]

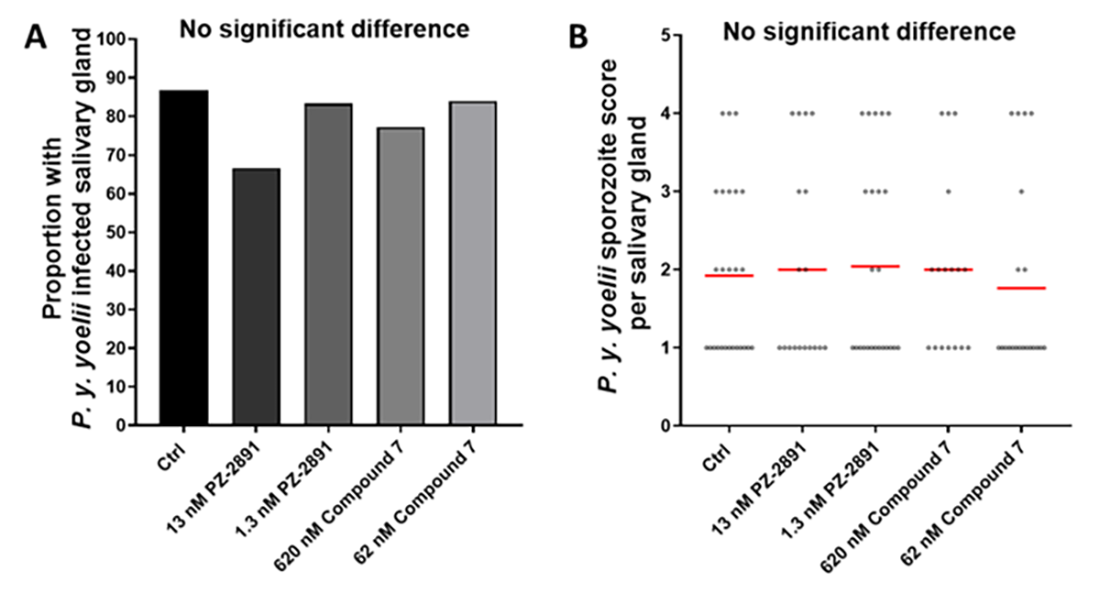

Supplement: Supplementary file 1 [file biomolecules-11-00807-s001.zip › Suppl Figure 2.tif]
